# Hepatitis A-associated jaundice, intermittent abdominal pain, and hepatic cystic mass: case report

**DOI:** 10.1093/jscr/rjaf113

**Published:** 2025-03-07

**Authors:** Alaa Aljohani, Nawaf Alashgar, Amal Alkhalaf, Shaden AlGhamdi, Mohammed Alsharit, Abrar Alageel

**Affiliations:** Surgery Department, Security Force Hospital, Al Riyadh, Saudi Arabia; Surgery Department, Security Force Hospital, Al Riyadh, Saudi Arabia; Surgical Oncology Department, King Fahad Medical City, Al Riyadh, Saudi Arabia; Prince Mohammed Bin Abdulaziz Hospital, Al Riyadh, Saudi Arabia; King Faisal Specialist Hospital and Research Center, Riyadh, Saudi Arabia; Surgery Department, Security Force Hospital, Al Riyadh, Saudi Arabia

**Keywords:** mucinous cystic neoplasm, hepatitis A

## Abstract

Mucinous cystic neoplasms of the liver are mostly benign but state challenging clinical problems. The case report emphasizes the need for thorough assessment and surgery intervention in patients with hepatic cystic masses in the context of potential biliary complications. A 21-year-old female patient presented with jaundice, minimal pruritus, and episodic right upper quadrant pain and a recent history of hepatitis A infection raised the initial concern. Imaging confirmed a multiseptated hepatic cystic mass with biliary communication, consistent with an MCN. The patient was treated with an exploratory laparotomy with successful enucleation of the cyst, preserving biliary anatomy. The patient has experienced an uneventful recovery, focused on the effectiveness of the multidisciplinary approach involving gastroenterology, hepatology, and surgery. Hence, comprehensive diagnostics, skilled surgical techniques, and personal treatment strategies are needed to understand the nature of long-term effects and risk factors for recurrence.

## Introduction

Hepatic mucinous cystic neoplasms (MCNs) are rare cystic hepatic lesions that predominantly occur in middle-aged women. They are often benign but can sometimes have malignant characteristics. Due to their complex presentation and potential for biliary communication with possible complications, they pose challenges both in diagnosis and management [1, 2]. The case highlights the importance of a comprehensive diagnostic evaluation, multidisciplinary collaboration, and minimally invasive surgical techniques to preserve hepatic parenchyma while achieving complete resection. This case report strictly adheres to the SCARE guidelines by providing a comprehensive and structured account of the patient’s clinical presentation, diagnostic work-up, surgical intervention, and post-operative course.

## Case presentation

### Patient details

A 21-year-old female patient presented with symptoms of jaundice, eyes, and urine, with only minimal pruritus without fever and periodic abdominal pain for the past month. Her medical history showed a recent episode of hepatitis A that was self-diagnosed one month before this presentation, otherwise, surgically and medically free. Physical examination revealed a soft and lax abdomen without tenderness or organomegaly. No other abnormalities were noted.

### Clinical findings

**Figure 1:**
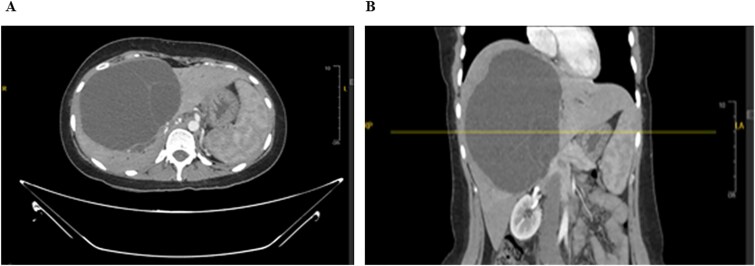
CT scan of the abdomen: Demonstrating the hepatic mass measuring involving the segments 4A, 4B, 5, 6, and 8. A) Transverse view, B) longitudinal view.

Laboratory studies were performed for serum alpha-fetoprotein (AFP), carcinoembryonic antigen (CEA), and carbohydrate antigen (CA). All the above investigations were normal except for an elevated level of CA-19.9. A computed tomography (CT) of the abdomen and chest with an intravenous contrast scan was performed. Chest imaging did not reveal any malignancy. Abdominal imaging demonstrated a big, multiseptated hepatic cystic mass, measuring 14 × 11 × 16 cm, involving segments 4A, 4B, 5, 6, and 8, shown in Fig. 1.

**Figure 2:**
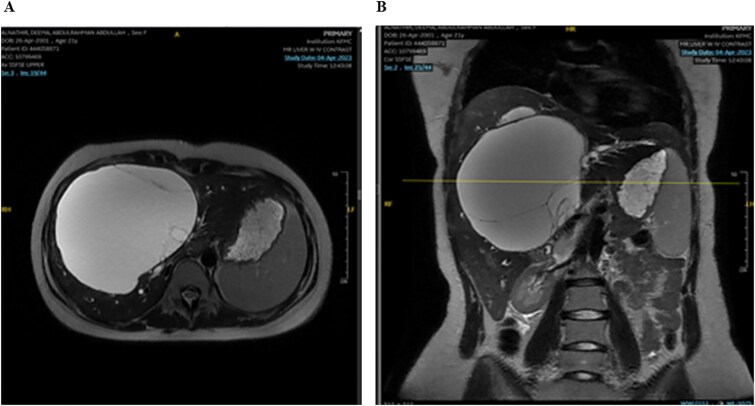
MRI of the liver: Demonstrating the mass in T2. Mild splenomegaly was noted without focal lesions. A) Transverse view, B) longitudinal view.

The mass had fine, minimally enhancing septations without any solid enhancing component. Communication with the right intrahepatic biliary ducts was observed, leading to moderate intrahepatic biliary duct dilatation. Magnetic resonance imaging (MRI) of the liver using contrast reveals a large hepatic cystic mass, corresponding to dimensions noted on CT, showing bright T2 signal intensity with thin septations without a solid component, illustrated in Fig. 2.

**Figure 3:**
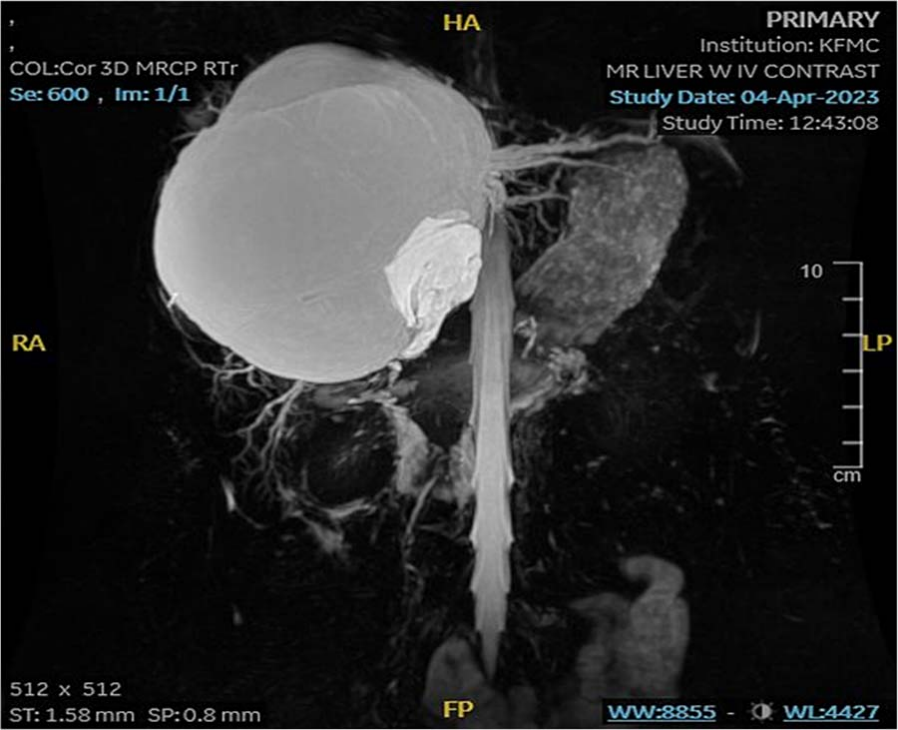
MRI of the liver: Demonstrating smaller bright T2 signal intensity lesions without post-contrast enhancement were observed in segment 7 and segment 6, consistent with cysts.

Mild splenomegaly (13.4 cm) with no focal lesions was noted, which exerted a mass effect on the patent hepatic vasculature. No osseous lesions or lymphadenopathies were identified. Three smaller bright T2 signal intensity lesions that did not enhance on post-contrast scanning were seen in segments 7 and 6, with measurements 0.2 × 0.1 cm, 0.7 × 0.7 cm, and 0.9 × 0.4 cm, respectively. The consistency of the cyst is evident from Fig. 3.

**Figure 4:**
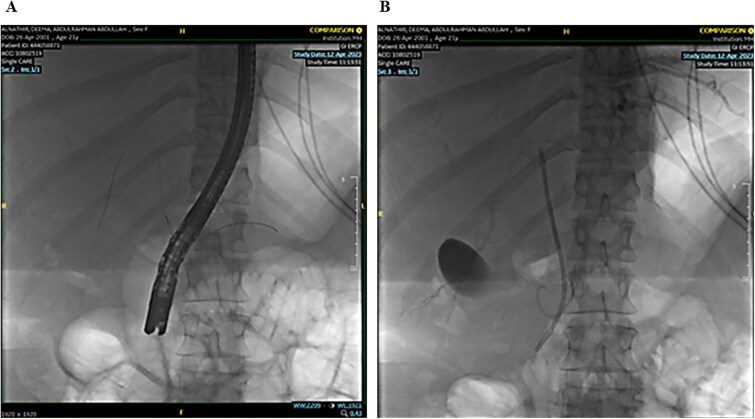
Endoscopic retrograde cholangiopancreatography (ERCP): Demonstrating the cannulation procedure and the placement of the cannula.

The remaining hepatic parenchyma had homogenous enhancement and smooth outlines. As shown in Fig. 4, the Endoscopic retrograde cholangiopancreatography (ERCP) was performed using double-guided wire and successfully resulted in bile duct cannulation.

**Figure 5:**
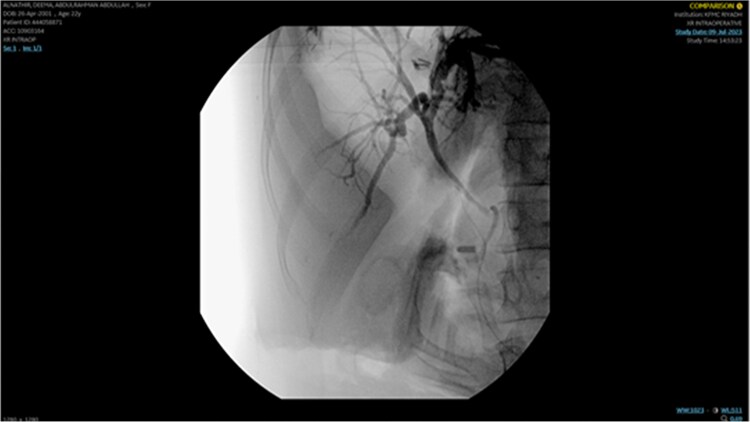
Demonstrating placement of a plastic stent in the right hepatic duct and ventral pancreatic duct as post-ERCP prophylaxis.

A single segmental biliary stricture was identified in the common hepatic duct. Biliary sphincterotomy was conducted as a form of post-ERCP prophylaxis, and a plastic stent was placed in the right hepatic duct and ventral pancreatic duct, as shown in Fig. 5. Visualization of the biliary tree confirmed the patency of the ducts.

### Surgical interventions

An exploratory laparotomy was performed, where aspiration and dissection of the hepatic cystic mass without requiring hepatectomy was allowed. Intraoperative aspiration of the cyst was performed without any spill. Partial dissection of the cyst wall on frozen analysis showed MCN. The cyst was successfully resected from the liver without communication to the biliary right system. Extensive adhesion was found to the segment four hepatic ducts, where primary repair using a monofilament polydioxanone suture (PDS suture) was done. Intraoperative cholangiogram showed good anatomy in both the right and the left systems up to the level of second-order biliary radicles. Omentoplasty was done to enhance the surgical repair further.

### Pathology

Histopathological examination of the resected specimen showed a multi-loculated cystic lesion lined by bland mucinous epithelium and a subepithelial hypercellular stroma resembling ovarian tissue. Minimal patchy atypia was seen, suggesting reactive changes. Immunostaining was done to confirm the biliary nature of the epithelium, which showed positivity for CK7. Intracellular mucin is confirmed with a special stain, DPAS. Stromal cells were positive for progesterone receptor (PR) and estrogen receptor (ER) immunostains. There was no presence of high-grade dysplasia or invasive carcinoma. The final diagnosis from the above findings was MCN. The cytological examination of the aspirated cyst fluid showed an acute neutrophilic inflammatory response similar to an abscess. Scattered giant multinucleated cells and macrophages were also seen. Fluid culture was negative for both bacteria and fungi.

### Post-operation

The patient tolerated the surgical intervention well, with no complications and stable vital signs. On the seventh postoperative day, the patient was discharged from the hospital. The patient did well at the most recent appointment without complaints, thus pointing out a successful recovery and positive outcome.

## Discussion

This case illustrates the rare presentation of a large, multiseptated MCN, emphasizing its diagnosis and management difficulties. Unlike common hepatic lesions, MCNs can involve biliary communication, complicating the surgical intervention and thus increasing the risk of complications [3, 4]. The presence of jaundice, irregular abdominal pain, and a recent infection of hepatitis A made the case quite specific. This is also characterized by its approach to preserving hepatic parenchyma using minimally invasive surgical techniques, such as enucleation, which consequently prevented radical hepatectomy. This underscores the importance of intraoperative cholangiography and frozen section analysis in creating an efficacy of precise tissue resection without much risk to the biliary system.

The study contributes to deepening the understanding of managing MCN and customized treatment plans set by real-time diagnostics and expert preparation for surgery. This case adds to the existing literature by offering insight into managing complex hepatic cystic lesions while emphasizing the importance of multidisciplinary collaboration [5].

## Conclusion

This case underlines the issue that led to the initial concern for a more aggressive pathology, having recently been affected by hepatitis A infection and jaundice, stressing the need for a thorough diagnostic workup of hepatic cystic lesions. The easy, successful surgical enucleation that reduced the need for radical hepatectomy and preserved the biliary system exemplifies the effectiveness of a minimally invasive approach for MCNs. The patient's uneventful postoperative course and successful recovery further emphasize the benefits of a multidisciplinary approach involving gastroenterology, hepatology, and surgery in ensuring optimal patient outcomes.
